# The multicomponent medication Spascupreel attenuates stress‐induced gut dysfunction in rats

**DOI:** 10.1111/nmo.13798

**Published:** 2020-02-14

**Authors:** Vassilia Theodorou, Catherine Beaufrand, Sophie Yvon, Guylaine Laforge, Yvonne Burmeister, Andrea Müller, Bernd Seilheimer, Lionel Bueno, Helene Eutamene

**Affiliations:** ^1^ INRA ToxAlim UMR 1331 Neuro‐Gastroenterology and Nutrition Group ENVT INP‐Purpan UPS Université de Toulouse Toulouse France; ^2^ Heel GmbH Baden‐Baden Germany

**Keywords:** gut‐brain axis, irritable bowel syndrome, multicomponent medication, partial restraint stress, Spascupreel, SP-11

## Abstract

**Background:**

Irritable bowel syndrome (IBS) is a common disorder worldwide. It is characterized by abdominal pain/discomfort and changes in bowel habits. Due to the multifactorial pathophysiology and the heterogeneity of IBS patients, appropriate treatment of IBS is still a challenge. Spascupreel (SP‐11), as a multicomponent medication, has the potential to modulate multiple pathophysiological pathways simultaneously. Therefore, the objective of the current study was to investigate the effects of oral SP‐11 treatment on stress‐induced changes of peripheral and central functions in a rat model mimicking human IBS.

**Methods:**

Naïve Wistar rats were treated with SP‐11 (0.9 tab/kg) or NaCl 0.9% by oral gavage for 4 days before 2‐hour partial restraint stress (PRS) procedure. Twenty minutes after PRS, central and peripheral stress‐induced changes affecting IBS were assessed. These include the hypothalamic‐pituitary‐adrenal (HPA) axis response through plasma ACTH and corticosterone measurements, visceral pain in response to colorectal distension, gut permeability, colonic mast cell number, and sensitization as well as gut transit time.

**Results:**

Treatment with SP‐11 reduced the HPA axis activation in response to PRS. At the gut level, a reduction in colonic hypersensitivity to colorectal distension, a normalization of gut transit time acceleration, a reduced mast cell sensitization, and a trend toward reduced gut hyperpermeability were observed.

**Conclusions:**

These data suggest that stress‐induced IBS signs can be reduced using SP‐11 in rats. The observed effects and the good tolerability of the drug make SP‐11 an innovative candidate in the management of IBS.

## Graphical abstract

In a stress‐induced rat model of irritable bowel syndrome (IBS), SP‐11 reduced the hypothalamic‐pituitary‐adrenal axis activation and positively modulated the gut function. Multiple pathophysiological pathways were beneficially targeted by SP‐11 leading to a reduction of IBS signs in rats.

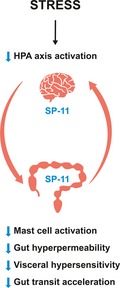

**Key Points**

- SP‐11 positively modulated the gut function.
- SP‐11 reduced the HPA axis activation.
- SP‐11 reduced IBS signs in a rat model.
- SP‐11 beneficially targeted multiple pathophysiological pathways associated with IBS.

## 1 INTRODUCTION

Irritable bowel syndrome (IBS) is one of the most common conditions that a physician faces in the gastrointestinal clinic. According to the Rome criteria IV, 5%‐7% of the general population suffers from IBS symptoms.^1^ Abdominal pain is a cardinal IBS symptom associated with changes in bowel habits (diarrhea and/or constipation) (for review Enck et al, 2016^2^). IBS patients are stratified into four subtypes according to the predominant changes in bowel habits: diarrhea‐predominant (IBS‐D), constipation‐predominant (IBS‐C), both diarrhea and constipation (IBS‐M) and unclassified (IBS‐U) (for review Enck et al, 2016^2^). The symptoms strongly affect the patient's quality of life^3^ and represent a significant socioeconomic burden.^4^ In the absence of a clear identification of organic features and lack of reliable biomarkers, the IBS pathophysiology is still not completely understood. IBS symptoms may originate from several peripheral and/or central mechanisms. Among them, dysfunctional gut‐brain axis,^5^ low‐grade intestinal inflammation,^6^ increase in mucosal mast cells,^7^ intestinal microbiota dysbiosis,^8^ and impaired intestinal barrier function^9^ have been reported in the literature. For instance, alterations in the central nervous system (CNS), caused by anxiety and stressful psychological stimuli, triggered abnormal gastrointestinal motility,^10^ heightened visceral sensations,^11^ and increased gut permeability.^12^

In order to understand the IBS pathophysiology, acute and chronic stress animal models mimicking IBS features, such as changes in visceral sensitivity, gut transit alterations, mast cell infiltration, and impaired intestinal barrier function, have been developed.^13^, ^14^ Indeed, in rats acute restraint stress is reflected by both an increase in adrenocorticotropic hormone (ACTH) and corticosterone plasma concentrations.^15^ This is associated with visceral hypersensitivity to colorectal distension (CRD), a central release of corticotrophin releasing factor (CRF),^13^ and an increase in gut permeability.^16^ Mast cells and their products play an important role in the pathophysiology of IBS.^17^ Uncontrolled or dysregulated mast cell activation may interfere with gut homeostasis, generate tissue dysfunction, and promote inflammation in diverse gastrointestinal diseases and functional gastrointestinal disorders.^18^ In rats, acute stress increases the colonic mast cell histamine content, a peripheral effect, mediated by the release in the cascade of interleukin‐1 (IL‐1) and CRF.^19^

Regarding the multifactorial pathophysiology and the heterogeneity of the IBS population, appropriate treatment of IBS is still a challenge. Therapeutic strategies are often limited to the treatment of symptoms with drugs focusing on motor/sensory abnormalities. For patients with psychological comorbidity, psychopharmacological agents can also be useful in the treatment of IBS.^20^

To target multiple pathophysiological pathways associated with IBS simultaneously, a multicomponent/multitarget‐based treatment might be suitable. SP‐11 is a multicomponent medicinal product, which was previously investigated in several clinical studies in conditions such as gastrointestinal cramps or spasmodic gastritis^21^, ^22^, ^23^; however, its mode of action is still poorly understood. In this study, we aimed to investigate the effects of SP‐11 on the multifactorial pathophysiological mechanisms of IBS.

## 2 MATERIALS AND METHODS

### 2.1 Test item

Spascupreel (SP‐11) tablets were manufactured by Heel GmbH, Germany, according to GMP standards. The study medication was packaged, shipped, and labeled by Heel GmbH, Germany. One tablet contains Citrullus colocynthis D4 30 mg, Ammonium bromatum D4 30 mg, Atropinum sulfuricum D6 30 mg, Veratrum album D6 30 mg, Magnesium phosphoricum D6 30 mg, Gelsemium sempervirens D6 30 mg, Passiflora incarnata D2 15 mg, Amanita muscaria D4 15 mg, Matricaria recutita D3 15 mg, Cuprum sulfuricum D6 15 mg, and Aconitum napellus D6 60 mg (D stands for 10× dilution of the mother tincture). The tablets were dissolved in water and administered at a dose of 0.9 tab/kg once daily at 9 am for 4 days by oral gavage in 1 mL of water using a gastro‐esophageal cannula.

### 2.2 Animals

According to the parameter studied, male or female Wistar rats (175‐225 g; Janvier SA) were used. Animals were individually housed in a temperature‐controlled room (21 ± 1°C) and maintained in a 12/12‐hour light/dark cycle. Rats had free access to water and were fed ad libitum with laboratory pellets (Envigo, Teklad Global Diet®). The Local Animal Care and Use Committee of Institut National de la Recherche Agronomique approved all experimental protocols (n° MP/01/53/10/11).

### 2.3 Experimental design

**Figure 1:**
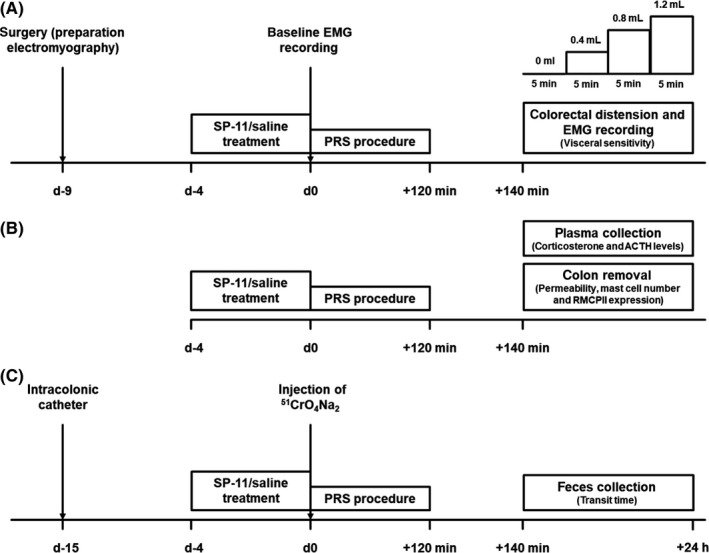
Experimental designs. A, In the first experiment, the effect of SP‐11 on partial restraint stress (PRS)‐induced visceral hypersensitivity to colorectal distension was evaluated. B, The second experiment aimed to investigate the effect of SP‐11 on stress‐associated changes of plasma ACTH and corticosterone levels as well as gut permeability, mast cell number, and activation (RMCPII expression). C, In the last experiment, the effect of SP‐11 on PRS‐induced gut transit time acceleration was analyzed

Three series of experiments were conducted. In all of them, SP‐11 (0.9 tab/kg) or NaCl 0.9% (1 mL/rat) were administered per oral gavage 4 days before 2‐hour partial restraint stress (PRS) session. Twenty minutes after PRS, ACTH, corticosterone, visceral hypersensitivity, gut permeability, gut transit time, mast cell number, and rat mast cell protease II (RMCPII) expression were investigated. In the first series, visceral sensitivity to colorectal distension was assessed by electromyography (EMG) recording in the same animals before (basal condition) and 20 minutes after the PRS session (Figure 1A). The second series was performed to assess the physiological stress response via measuring plasma corticosterone and ACTH concentrations. In addition, after removal of colonic segments, paracellular permeability was assessed, and mast cell number and RMCPII expression were measured (Figure 1B). The third series aimed to investigate gut motility by measuring the transit time (Figure 1C).

### 2.4 PRS procedure

All stress sessions were performed at the same time range of the day (between 10 am and 12 pm) to minimize any influence of circadian rhythms. Stress effects were studied using a single 2‐hour session of PRS which is considered as a mild non‐ulcerogenic model.^24^ Under light anesthesia with ethyl ether, upper forelimbs were taped up to the thoracic trunk in order to constrain animal body movements. Rats were then replaced in their home cages for 2 hours.

### 2.5 Visceral sensitivity in response to CRD

To evaluate abdominal contractions, an index of visceral sensitivity, female rats were surgically prepared for EMG and equipped with three groups of NiCr wire electrodes (Sandvik). These were implanted into the abdominal external oblique muscle 5 days prior to the experiment. The myoelectrical activity was recorded by EMG as previously described by Morteau et al, 1994.^25^ Rats were placed in a polypropylene tunnel and a balloon consisting of an arterial embolectomy catheter (Fogarty®; Edwards Laboratories Inc) was slowly placed 4 cm into the rectum and taped at the base of the tail. The balloon was progressively inflated by steps of 0.4 mL, from 0 to 1.2 mL, each step lasting 5 minutes. Data are presented as the number of abdominal contractions/5 minutes.

### 2.6 Gut permeability

Twenty minutes after the stress procedure, male rats were lethally anaesthetized (pentobarbital sodium 100 mg/kg ip (Ceva)). Immediately after sacrifice, sections of the colon, cut along the mesenteric border, were mounted in Ussing chambers (Easymount). All segments were properly adjusted to fit the entire exposed tissue surface area corresponding to 0.5 cm^2^. Chambers were filled with 5 mL of Krebs‐Henseleit solution (Sigma) maintained at 37°C and continuously gassed with 95% O_2_/5% CO_2_. Transepithelial resistance (TER) was monitored throughout the experiment to assess the viability of the tissue.^26^ Paracellular permeability was assessed by measuring mucosal‐to‐serosal flux of fluorescein isothiocyanate (FITC)‐labeled 4 kDa dextran (Sigma). After 10‐minute equilibrium period, 500 µL of the Krebs‐Henseleit solution was replaced by 500 µL of the FITC‐labeled 4 kDa dextran (2.2 mg/mL as the final concentration) in the mucosal side. After 1 hour, fluorescence was measured in the serosal buffer side by a fluorimeter (wavelength of 540 nm, Tecan). Results were expressed as the flux of dextran crossing the epithelial barrier (nmol/cm^2^/h).

### 2.7 Transit time

Under general anesthesia (acepromazine 0.6 mg/kg ip (Calmivet, Vetoquinol) and ketamine 120 mg/kg ip (Imalgene, Rhone Merieux)), male rats were equipped with an intracolonic catheter (Folioplast) and accustomed to eat 3 hours per day during 15 days. Then, the animals received 1 µCi of ^51^CrO_4_Na_2_ (Perkin Elmer Life Sciences) dissolved in 0.1 mL of saline by intracolonic route and placed on a conveyor belt supporting collector tubes changed every 60 minutes. The excretion of ^51^CrO_4_Na_2_ was collected for each hour during 24 hours and was analyzed with a gamma counter (Cobra II; Packard, Meriden, CT, USA). The mean retention time (MRT) was calculated using the following formula:

$$\text{MRT}\left(h\right)=\left(t_{5}+t_{15}+\ldots \ldots \ldots \ldots .t_{95}\right)/100.$$

*t*_5_, *t*_15_....*t*_95_ corresponds to times for which 5, 15.....0.95% of the marker was excreted in the feces.

### 2.8 Colonic mast cell numbers and RMCPII expression

Distal colon specimens were collected from male rats and fixed in 4% buffered formalin and incubated 24 hours in 30% sucrose at 4°C. Samples were embedded in Neg50 medium (Microm) and frozen in isopentane at −45°C. Cryostat sections (7 µm) were postfixed with acetone (10 minutes, −20°C) and hydrated in PBS‐Tween. After incubation in blocking solution (PBS containing 1% bovine serum albumin and 2% donkey serum), sections were incubated with sheep anti‐RMCPII antibodies (overnight, 4°C, 1/500) (Moredun) followed by incubation with Alexa Fluor® 594 donkey antisheep IgG (1 hour 30 minutes, room temperature, 1/2000) (Life Technologies). After each incubation period, sections were rinsed in PBS‐Tween. Sections were mounted in ProLong® Gold Antifade Mountant (Life Technologies) and examined under a Nikon 90i fluorescence microscope (Nikon). Mast cell number per mm^2^ of mucosa and the intensity of RMCPII within mast cells were quantified using Nikon‐Elements‐Ar software. For each animal, five fields of view were counted.

### 2.9 Plasma ACTH and corticosterone levels

After sacrifice (pentobarbital sodium 100 mg/kg ip), blood for ACTH and corticosterone levels was collected from the abdominal aorta into EDTA‐containing Vacutainer® tubes (Sigma). The freshly drawn blood was centrifuged at 2000 *g* for 15 minutes at 4°C. Plasma was collected and subsequently stored at −80°C until used for ACTH and corticosterone determination. Quantification of plasma ACTH was performed using the ACTH (mouse/rat) ELISA kit (Tebu‐bio), and corticosterone was evaluated using the IDS corticosterone EIA kit (Immunodiagnostic System) according to the manufacturer's instructions. Plasma corticosterone is expressed in µg/mL and plasma ACTH is shown in ng/mL.

### 2.10 Statistical analysis

For each parameter studied, data were expressed as mean ± SEM. For statistical analysis, Prism 4.0 (GraphPad) was used. Comparisons between the different groups were performed using an analysis of variance (one‐way ANOVA) followed by a Bonferroni's post‐test. A value of *P* < .05 was considered as statistically significant, and a value between *P* = < .05 to *P* < .1 was considered as a trend toward statistical significance.

## 3 RESULTS

### 3.1 Visceral sensitivity

**Figure 2:**
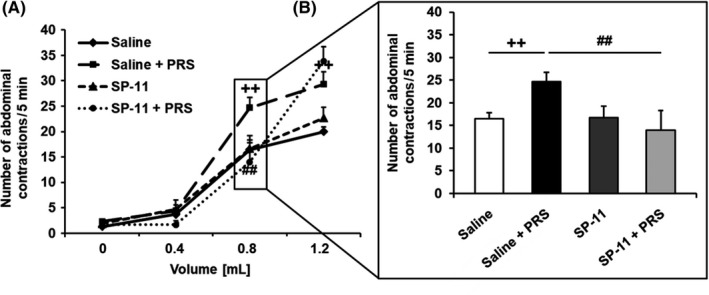
Influence of SP‐11 on stress‐induced visceral hypersensitivity to colorectal distension. A, Partial restraint stress (PRS) increased the abdominal contractions in comparison with non‐stressed saline‐treated animals (saline group: n = 11 and saline + PRS group: n = 10). SP‐11 had no effect on visceral sensitivity in basal conditions (SP‐11 group: n = 9), but significantly reduced the PRS‐induced visceral hypersensitivity (SP‐11 + PRS group: n = 9). B, Inhibitory effect of SP‐11 on visceral hypersensitivity induced by stress at the distending volume of 0.8 mL. ^++^ *P* < .01 comparing non‐stressed and stressed saline‐treated rats. ^##^ *P* < .01 comparing stressed animals treated with saline or SP‐11

Colorectal distension increased the frequency of abdominal contractions per 5 minutes in a volume‐dependent manner in all rats. Comparing non‐stressed (basal conditions) and stressed animals treated with saline, the first volume of distension that significantly increased the number of abdominal contractions was 0.8 mL, indicating a visceral hypersensitive response (16.45 ± 1.33 vs 24.70 ± 2.00 number of abdominal contractions/5 minutes, respectively, *P* < .01; Figure 2A). Therefore, 0.8 mL was considered as a reference volume to evaluate the effect of SP‐11 on visceral sensitivity to CRD. SP‐11 induced a significant inhibitory effect on stress‐induced visceral hypersensitivity response (24.70 ± 2.00 vs 14.00 ± 4.29 number of abdominal contractions/5 minutes, respectively, *P* < .01), but had no impact on visceral sensitivity in response to CRD in non‐stressed animals (16.45 ± 1.33 vs 16.67 ± 2.58 number of abdominal contractions/5 minutes, respectively; Figure 2B).

### 3.2 Intestinal permeability

**Figure 3:**
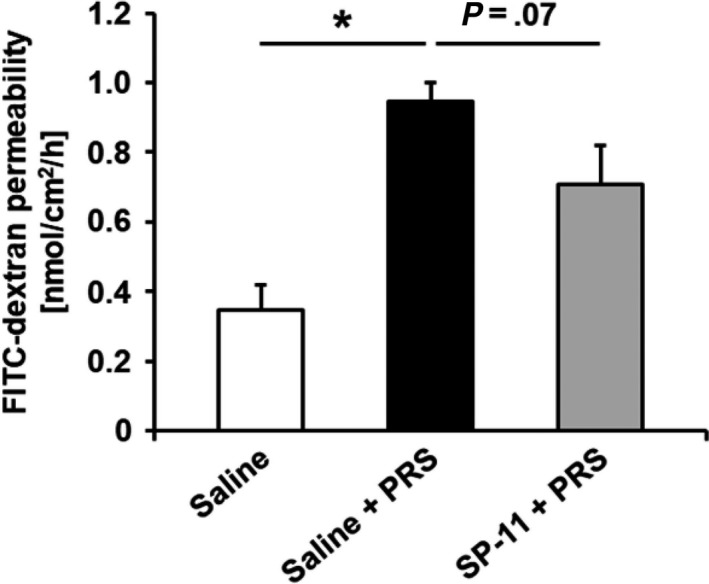
Effect of SP‐11 on intestinal hyperpermeability induced by stress. Partial restraint stress (PRS) increased intestinal FITC‐dextran permeability in comparison with non‐stressed animals treated with saline (saline group: n = 8 and saline + PRS group: n = 9, *P < .05). SP‐11 treatment showed a trend toward reduced gut hyperpermeability of stressed animals (SP‐11 + saline group: n = 8, *P* = .07)

Partial restraint stress significantly increased the FITC‐dextran permeability in colonic segments compared with non‐stressed saline‐treated rats (0.35 ± 0.07 vs 0.95 ± 0.05 nmol/cm^2^/h, respectively, *P* < .05). SP‐11 showed a trend toward attenuating the colonic hyperpermeability induced by PRS (0.95 ± 0.05 vs 0.71 ± 0.11 nmol/cm^2^/h, respectively, *P* = .07; Figure 3).

### 3.3 Gut transit

**Figure 4:**
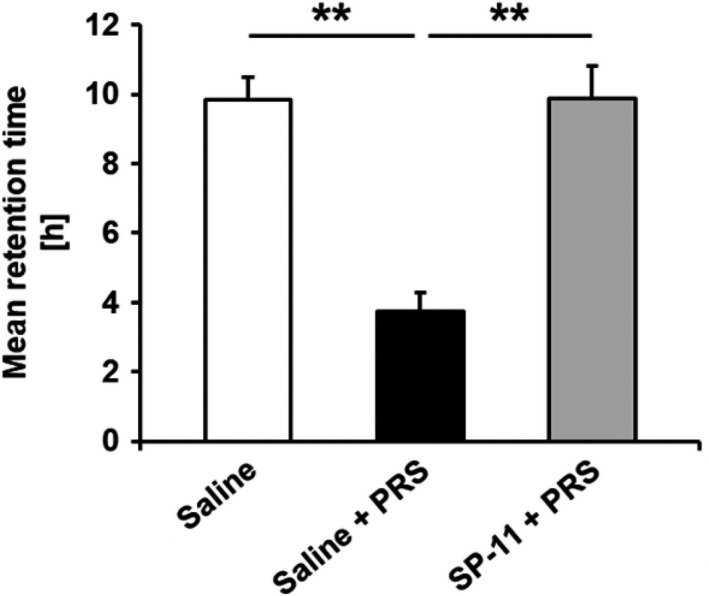
Effect of SP‐11 on gut intestinal transit. Partial restraint stress (PRS) accelerated intestinal transit in comparison with saline‐treated non‐stressed rats (saline and saline + PRS group: n = 10, **P < .01). SP‐11 reduced stress‐induced gut transit acceleration (SP‐11 + PRS group: n = 10, **P < .01)

After stress induction, colonic mean retention time significantly decreased in comparison with non‐stressed animals treated with saline (9.85 ± 0.65 vs 3.75 ± 0.52 hours, respectively, *P* < .01). Pretreatment with SP‐11 significantly suppressed stress‐induced colonic transit acceleration (3.75 ± 0.52 vs 9.89 ± 0.91 hours, respectively, *P* < .01; Figure 4).

### 3.4 Mast cells

**Figure 5:**
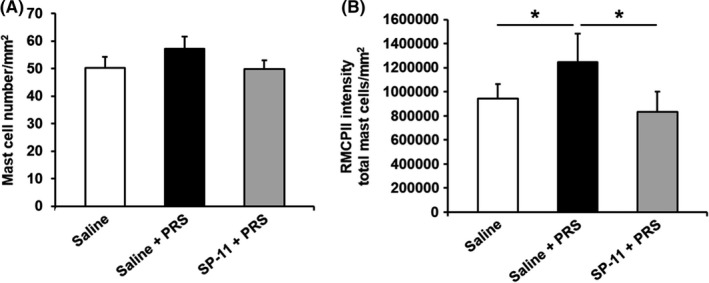
Influence of SP‐11 on mast cell number and activation. A, Partial restraint stress (PRS) did not affect mast cell number (saline and saline + PRS group: n = 10 and SP‐11 + PRS group: n = 9); B, but induced an increase in intracellular RMCPII expression compared with saline‐treated non‐stressed rats (saline and saline + PRS group: n = 7, *P < .05). SP‐11 significantly reduced the RCMPII expression (SP‐11 + PRS group: n = 7, *P < .05)

Compared with non‐stressed saline‐treated animals, PRS significantly increased RMCPII expression in mast cells (941 676.93 ± 120 476.49 vs 1 244 611.35 ± 238 132.58 total mast cells/mm^2^, respectively, *P* < .05), without affecting the colonic mast cell number (50.23 ± 4.08 vs 57.17 ± 4.45 mast cell number/mm^2^, respectively). Pretreatment with SP‐11 had also no influence on colonic mast cell numbers (57.17 ± 4.45 vs 49.78 ± 3.22 mast cell number/mm^2^, respectively), but significantly reduced the RMCPII levels in stressed rats (1 244 611.35 ± 238 132.58 vs 830 665.39 ± 169 482.81 total mast cells/mm^2^, respectively, *P* < .05; Figure 5A,B).

### 3.5 Corticosterone and ACTH levels

**Figure 6:**
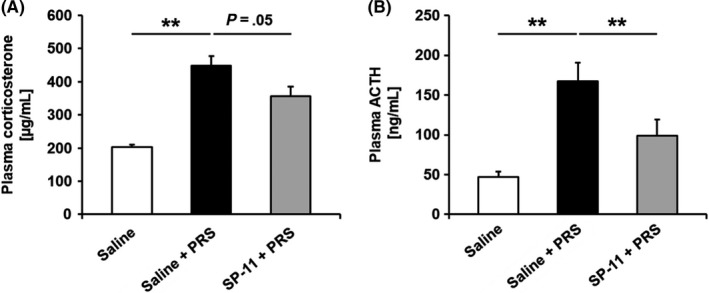
Effect of SP‐11 on plasma corticosterone and ACTH levels. A and B, Partial restraint stress (PRS) increased both corticosterone and ACTH plasma levels compared with non‐stressed animals treated with saline (saline and saline + PRS group: n = 10, **P < .01). SP‐11 treatment showed a trend toward reduced plasma corticosterone levels and significantly reduced the plasma ACTH concentration (SP‐11 + PRS group: n = 10 for ACTH, ^**^ *P* < .01 and n = 9 for corticosterone, *P* = .05)

Partial restraint stress led to a significant increase in plasma corticosterone levels compared with non‐stressed saline‐treated rats (202.69 ± 8.43 vs 448.76 ± 28.30 µmol/mL, respectively, *P* < .01). SP‐11 showed a trend toward reduced corticosterone levels (448.76 ± 28.30 vs 355.78 ± 29.58 µmol/mL, respectively, *P* = .05; Figure 6A).

PRS also resulted in an increase of plasma ACTH levels in comparison with non‐stressed saline‐treated rats (46.66 ± 6.76 vs 167.43 ± 23.56 nmol/mL, respectively, *P* < .01). SP‐11 significantly reduced the stress‐induced increase in plasma ACTH level (167.43 ± 23.56 vs 98.52 ± 20.72 nmol/mL, respectively, *P* < .01; Figure 6B).

## 4 DISCUSSION

This study shows that a short‐term pretreatment (4 days prior to stress exposure) with SP‐11 reduced the HPA axis response to acute stress in rats as demonstrated by a significant decrease in plasma ACTH and a trend toward reduced corticosterone levels. Further, at the peripheral level, SP‐11 significantly reduced stress‐induced activation of mast cells, as indicated by the decrease in intracellular RMCPII expression, significantly reduced the visceral hypersensitivity as well as gut transit acceleration, and showed a trend toward reduced gut hyperpermeability.

There are no previously published studies exploring the mode of action of SP‐11, although a few clinical reports document its use for gastrointestinal symptoms with referencing an expected action on the smooth musculature.^23^, ^27^ We speculate that the mode of action will be a rather complex “network pharmacology”^28^ of potentially hundreds of targets covering immune, endocrine, smooth muscle and neural molecular interactions. The present study is the first attempt to characterize this medicinal product comprehensively in a model of IBS.

The effects of SP‐11 were first assessed by focusing on the two major features of IBS pathophysiology, visceral hypersensitivity, and impaired intestinal barrier function. SP‐11 showed a trend toward reduced stress‐induced gut hyperpermeability and significantly reduced visceral pain. The visceral hypersensitivity observed in IBS is defined as an enhanced perception of mechanical triggers (pressures of volumes) applied to the gut. The pioneer work of Ritchie (1973)^29^ reported for the first time the enhanced painful response of IBS sufferers resulting from rectal distension. Later, other clinical studies confirmed that the increased pain perception or discomfort in response to rectal distension is an important clinical feature in the majority of IBS patients.^30^, ^31^ The origin of visceral hypersensitivity is not fully understood yet, but central (stress, anxiety, depression) and/or peripheral factors (increased gut permeability, low‐grade inflammation, mucosal mast cell activation, and dysbiosis) are discussed.^32^ A visceral hypersensitivity in response to colorectal distension was also reported in animal models of stress.^13^, ^16^ The central origin of the stress‐induced visceral hypersensitivity was attributed to CRF and the peripheral one to colonic mast cell degranulation and increased gut permeability.^13^, ^16^ Despite the debate attributing the increase in gut permeability mostly to IBS‐D patients,^33^ Piche et al (2009)^9^ showed that increased intestinal paracellular permeability is a common characteristic of all IBS subtypes. This increased gut permeability is reflected by alterations in the tight junction complex. In IBS patients increased claudin 2 protein expression,^34^ downregulation of ZO‐1^9^ and occludin expression^35^, ^36^ as well as redistribution of claudin 1^36^ were observed. In animal models of stress, an increase in gut permeability was also described^16^, ^37^ resulting from colonocyte myosin light chain (MLC) phosphorylation leading to cytoskeleton contraction and subsequent tight junction opening as well as from occludin and JAM‐A down‐regulation.^38^ Further, a cause‐effect relationship between stress‐induced gut hyperpermeability and visceral hypersensitivity was shown in rats.^16^ Interestingly, in IBS patients a positive correlation between increased intestinal permeability and visceral pain was also reported.^39^ The epithelial barrier impairment observed in both humans and animals may result from an uptake of luminal contents (antigens, microbial patterns, etc) able to activate the mucosal immunity and release of mediators, which, in turn, sensitize afferent neurons leading to visceral hypersensitivity.^40^ Another major player able to be activated by stress and, in turn, release mediators impairing the epithelial barrier integrity is a mast cell. The study from Vanuytsel et al (2014)^12^ in humans clearly showed that increased small bowel permeability induced by acute psychological stress or CHR administration was prevented by co‐administration of disodium cromoglycate (a mast cell stabilizer). Besides the effect of mast cell mediators on intestinal epithelial barrier, they may also play a role in visceral sensitivity. For example, Wang et al (2014)^41^ showed that histamine and mast cell protease II released by mast cells may diffuse in a paracrine manner and sensitize enteric nerve terminals leading to enhanced sensitivity of spinal afferents. Similarly, Barbara et al (2007)^42^ demonstrated that mediators from IBS patients strongly excite rat nociceptive sensory nerves in the dorsal root ganglia. SP‐11 did not modify colonic mast cell numbers but significantly inhibited mast cell activation as reflected by the reduction in the RMCPII immunostaining. This finding is in agreement with a previous study showing that an acute stress in rats induces activation of colonic mast cells as reflected by intracellular increase in histamine content without modification of the mucosal mast cell number.^19^ Interestingly, according to these data, SP‐11 reduced the HPA axis response to stress by significantly reducing plasma levels of ACTH and showing a trend toward reduced corticosterone levels. Further, SP‐11 also reduced peripheral stress manifestations such as colonic mast cell activation by reducing their content in proteases (RMCPII), well described as a pronociceptive mediator,^43^ and gut transit. All these results clearly show that the SP‐11 treatment affects the gut‐brain axis regulation in the model of stress‐induced IBS used herein. Dysregulation of the gut‐brain axis in IBS is known to depend on several factors. Among these factors, stress plays a crucial role.^44^, ^45^ In the stress‐exposed gut‐brain axis, the main effector is the HPA axis and the activation of the autonomic nervous system, which is responsible for the peripheral manifestations of stress. Concerning the HPA axis response to stress, IBS patients infused by CRH responded with greater increase in both ACTH and cortisol suggesting a hyperresponsiveness of the stimulated HPA axis.^44^ In animals, similar observations were documented. Indeed, rodents submitted to acute or chronic stress show an increase in corticosterone and ACTH plasma levels and central CRH‐positive neurons^46^, ^47^ as well as peripheral increase in CRF receptor expression,^48^ confirming the relevance of animal models of stress in the investigation of IBS pathophysiology.

The peripheral manifestation of stress includes also gut motility disturbances. IBS patients exhibit an increased colonic and small intestine motor response to stress when compared to healthy subjects.^49^ In rodent models, the abnormal gut motor pattern was also documented several years ago.^50^ Indeed, an acceleration of gastrointestinal transit was shown using the restraint stress model^51^ and confirmed later by others using other models of stress (water avoidance stress, early life trauma)^52^, ^53^ or by CRF administration mimicking the stress effects.^54^ In this study, the stress‐induced acceleration of the gut transit was significantly reduced by SP‐11.

Taken these findings altogether, the following mode of action is proposed: SP‐11 reduces the stress‐induced HPA axis activation and dampens stress‐induced mast cell activation, preventing in turn intestinal epithelial barrier disruption and visceral hypersensitivity. The reduction in the HPA axis response to stress by SP‐11 probably leads also to a reduced autonomic nervous system activation resulting in a normalization of gut transit acceleration. The prevention of visceral hypersensitivity may result from a reduced direct activation of sensory nerve endings by mast cell mediators or by an indirect effect on the sensory nerve sensitization related to reduced luminal content upload and pro‐inflammatory mediator release by the mucosal immunity. Vice versa, the reduced HPA axis activation could also be a result of an effect of SP‐11 at the gut level, as the brain and gut communicate in a complex bidirectional way.

In conclusion, these data demonstrate the potential for SP‐11 in the treatment of IBS. After oral administration, the HPA axis showed a significantly reduced activation in terms of ACTH and a trend toward reduced corticosterone levels. Moreover, a significantly reduced visceral sensitivity to colorectal distension and mast cell activation, a significant normalization of the gut transit time, and a trend toward reduced gut permeability were shown. The observed effects and the good tolerability of the drug^23^ make SP‐11 an innovative candidate in the management of IBS.

## CONFLICTS OF INTEREST

YB, AM, and BS are employees of Heel.

## AUTHOR CONTRIBUTIONS

VT drafted the manuscript; CB, SY, and GF performed the experiments and analyzed the data; YB contributed to the interpretation of the results; AM critically reviewed and revised the manuscript; BS designed the study and critically reviewed and revised the manuscript; LB designed the study and interpreted the data, and HE analyzed and interpreted the data and drafted the manuscript.
